# Short-term results in a population based study indicate advantage for minimally invasive rectal cancer surgery versus open

**DOI:** 10.1186/s12893-024-02336-z

**Published:** 2024-02-10

**Authors:** Josefin Petersson, Peter Matthiessen, Kaveh Dehlaghi Jadid, David Bock, Eva Angenete

**Affiliations:** 1 Department of Surgery, SSORG Sahlgrenska University Hospital/Östra, 416 85 Göteborg, Sweden; 2 Sunshine Coast University Hospital, Britinya, QLD Australia; 3 Department of Surgery, Faculty of Medicine and Health Sciences, Örebro University, Örebro, Sweden; 4 Department of Surgery, Region Västra Götaland, Sahlgrenska University Hospital, Göteborg, Sweden

**Keywords:** Rectal cancer, Minimally invasive surgery, Laparoscopic surgery, Robotic surgery

## Abstract

**Background:**

The aim of this study was to determine if minimally invasive surgery (MIS) for rectal cancer is non-inferior to open surgery (OPEN) regarding adequacy of cancer resection in a population based setting.

**Methods:**

All 9,464 patients diagnosed with rectal cancer 2012–2018 who underwent curative surgery were included from the Swedish Colorectal Cancer Registry. Primary outcomes: Positive circumferential resection margin (CRM < 1 mm) and positive resection margin (R1). Non-inferiority margins used were 2.4% and 4%. Secondary outcomes: 30- and 90-day mortality, clinical anastomotic leak, re-operation < 30 days, 30- and 90-day re-admission, length of stay (LOS), distal resection margin < 1 mm and < 12 resected lymph nodes. Analyses were performed by intention-to-treat using unweighted and weighted multiple regression analyses.

**Results:**

The CRM was positive in 3.8% of the MIS group and 5.4% of the OPEN group, risk difference -1.6% (95% CI -1.623, -1.622). R1 was recorded in 2.8% of patients in the MIS group and in 4.4% of patients in the OPEN group, risk difference -1.6% (95% CI -1.649, -1.633). There were no differences between the groups in adjusted unweighted and weighted analyses. All analyses demonstrated decreased mortality and re-admissions at 30 and 90 days as well as shorter LOS following MIS.

**Conclusions:**

In this population based setting MIS for rectal cancer was non-inferior to OPEN regarding adequacy of cancer resection with favorable short-term outcomes.

## Background

Minimally invasive surgery (MIS) for rectal cancer is widely used with well recognized improved short-term outcomes such as less postoperative pain, less blood loss, faster recovery and shorter hospital stay when compared to open surgery (OPEN) [1–4]. However, randomised controlled trials have reported conflicting results with regard to the oncological outcomes of the two surgical techniques. The initial randomized trials comparing MIS and OPEN demonstrated similar short- and long-term oncological outcomes [5–7]. Two more recent randomised controlled trials were not able to demonstrate non-inferiority for MIS compared to OPEN for rectal cancer with regard to the composite outcome ‘successful resection’ [8, 9]. Neither study was powered to show non-inferiority for the two year follow up outcomes but no significant difference in rates of local recurrence, disease free or overall survival were reported. The latest published randomised controlled trial compared MIS with OPEN surgery for low rectal cancer and found similar short-term pathological and surgical outcomes [10]. Meta-analyses and systematic reviews have also reported diverging results regarding non-inferiority when comparing adequacy of cancer resection following MIS and OPEN surgical technique for rectal cancer [3, 11, 12]. Large cohort and population based studies have however supported the oncological safety in the use of MIS compared to OPEN both the adequacy of cancer resection and long-term oncological outcome [13–16].

The use of MIS for rectal cancer has increased in Sweden with 26% of elective resections performed using MIS in 2014 and 63% in 2018. A substantial and increasing part of all rectal cancer resections have been performed using robotic assisted laparoscopic technique (ROBOT) in Sweden, 9% in 2014 and 38% in 2018. It has been argued that ROBOT has potential technical benefits including the use of 3D imaging and articulating instruments. However, none of these advantages have shown to improve adequacy of cancer resection or long-term oncological outcomes when compared to OPEN and laparoscopic surgery [17–19]. Neither has any difference been found when comparing ROBOT to conventional laparoscopic surgery with regard to conversion rate, with the drawback of longer operating times [19, 20].

Furthermore, the results reported from randomised controlled trials reflect the oncological outcomes when operated on by colorectal surgeons highly specialized in minimally invasive surgery. Thus, little is known of the oncological outcomes when comparing MIS and OPEN surgery for rectal cancer in a standard care setting.

The objective of this study with a non-inferiority design was to determine if MIS for rectal cancer is non-inferior to OPEN for adequacy of cancer resection in routine healthcare by using data from high quality population based registries, including all tumour stages undergoing curative surgery. Secondary objectives were to compare short-term mortality and morbidity between the groups.

## Methods

### Study population and variables

In this population based study data was retrieved from the Swedish Colorectal Cancer Registry (SCRCR) with 98.8% completeness for rectal cancer and combined with data from the National Patient Registry (NPR) based on the unique Swedish identification number for outcomes on 30- and 90-day mortality and re-admission [21]. The NPR registers all in- and out-patient health care from 2001 onwards, and the reliability of the registry has been deemed as high through external validation [22].

The study was performed in accordance with the Declaration of Helsinki. Ethics approval was obtained from the Swedish Ethical Review Authority in Uppsala, Dnr 2018/129 and Dnr 2019–01787. Informed consent was waived as the study is an observational study using prospectively recorded registry data.

All patients diagnosed with rectal cancer from 1st of January 2012 up until 31st of December 2018 who subsequently underwent abdominal resection with curative intent were retrieved from the SCRCR. All cancer stages were included as long as curative intent was intended. Locally resected rectal cancers were not included.

Primary outcome was positive CRM and R1 resection. In the SCRCR, positive CRM is defined as tumour < 1 mm from resection margin and R1 resection is defined as tumour cells in the margin of resection. Patients with resection margins recorded in the SCRCR as “uncertain” or “unable to comment” or “missing” were all defined as missing in the analyses. Secondary outcomes were 30- and 90-day mortality, anastomotic leak and re-operation within 30 days, 30- and 90-day re-admission, LOS, positive distal resection margin (DRM) defined as < 1 mm and less than 12 lymph nodes in the surgical specimen as reported in the SCRCR and NPR. The conversion rate was recorded in the MIS group. Anastomotic leak has no formal definition in the SCRCR, but is nearly always a clinical anastomotic leak and is reported in the registry using a checkbox (yes/no) alternative. All data in the SCRCR is reported and recorded electronically.

In the adjusted analyses the type of surgery, the American Society of Anesthesiologists classification (ASA), age, body mass index (BMI), sex and clinical tumour stage (cTNM) were considered confounding variables for short-term mortality, morbidity and LOS. Type of surgery included anterior resection (AR), abdominoperineal resection (APR) and Hartmann’s procedure. Year of surgery was also deemed to be a potential confounding variable and adjusted for in the analyses. The outcomes CRM, R1 and DRM were further adjusted for cT stage, cN stage, presence of vascular and perineural invasion indicating more advanced tumours. Where cT and cN were missing yp/pT and yp/pN were used.

Conventional laparoscopic (LAP) and robot assisted laparoscopic surgery (ROBOT) were analysed as one group (MIS). ROBOT has been registered in the SCRCR since 2014 and a subgroup analysis was performed comparing ROBOT and OPEN between 2014 to 2018.

### Statistical analysis

A statistical analysis plan was agreed upon prior to analysing data. Analyses were performed according to the intention-to-treat principle. Baseline characteristics of patients were presented as frequencies and percentages or median with interquartile range (IQR). Differences between groups were reported with p-values using Chi-square test for categorical variables and Mann–Whitney U test for continuous variables. In the multiple regression analyses the following variables were adjusted for: type of surgery, ASA class, age, BMI, sex, cTNM and year of surgery. CRM, R1 and DRM were further adjusted for: cT stage, cN stage, proportion of vascular- and perineural invasion. Binary outcomes were analysed using logistic regression and continuous outcomes were log transformed before linear regression was performed. A second inverse probability treatment weighted analysis was performed using propensity scores. Propensity scores were determined using a logistic regression model adjusting for previously identified potential confounders. Non-inferiority was assessed by risk difference analyses with 95% confidence intervals using the predefined non-inferiority margin of 2.4% for CRM as suggested by the Delphi consensus consisting of rectal cancer experts worldwide and for R1 we used the cumulative figure suggested for CRM and DRM 4% [23]. All analyses were performed using SPSS Statistics 25.0 ®.

## Results

### Study population

**Fig. 1:**
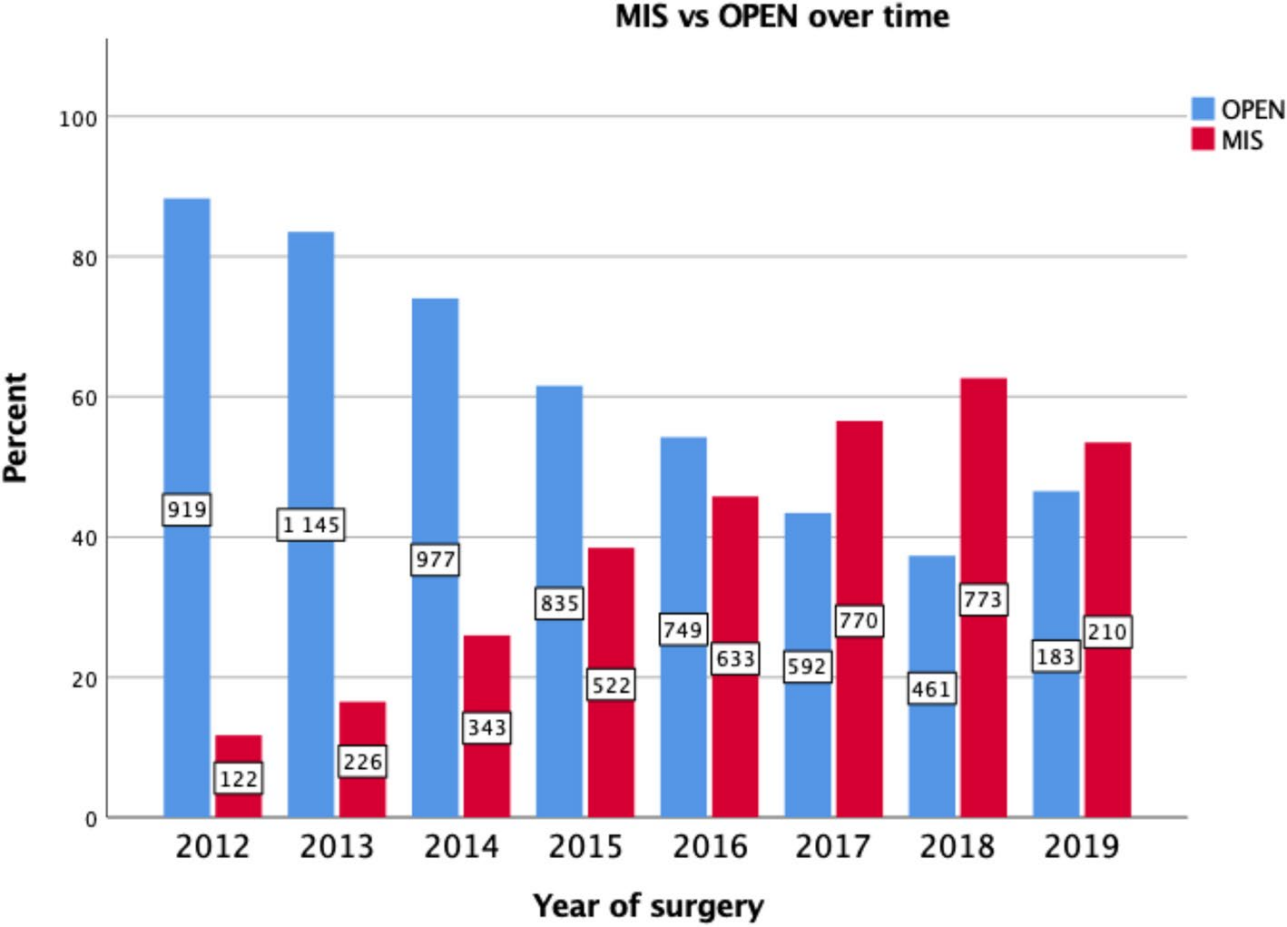
Number of procedures with percentage of MIS and OPEN per year

A total of 9464 patients diagnosed 2012–2018 with rectal cancer and receiving resection surgery with curative intent were included from the SCRCR. MIS was performed in 38% of cases, the proportion of patients undergoing MIS increased over the time period and the conversion rate decreased from 20% in 2012 to 12% in 2019 (Fig. 1). The OPEN group included a higher proportion of male patients and a higher proportion of ASA III-IV patients compared to the MIS group (Table 1). The OPEN group displayed more advanced clinical T stage, N stage and TNM stage including more high-grade tumours and a higher proportion of patients had received neoadjuvant treatment compared to the MIS group (Tables 1 and 2). The groups were not statistically significantly different with regard to age, BMI, tumour height, vascular invasion or perineural invasion (Tables 1 and 2). APR represented 38.5% of resections in both groups but Hartmann’s procedure was more frequently performed in the OPEN group (Table 2). A higher proportion of MIS underwent AR compared to the OPEN group and diverting ileostomies were more common in the MIS group (*P* < 0.001) (Table 2). Intraoperative bowel perforations were more frequent in the OPEN 5.1% vs. MIS 3.7% but perforations close to the tumour did not differ between the two groups (*P* = 0.134) (Table 2). The MIS group demonstrated statistically significantly less bleeding but longer operating times compared to the OPEN group (Table 2).

**Table 1:** Baseline characteristics of patients who were diagnosed with rectal cancer in Sweden 2012–2018 and underwent surgical resection MIS vs OPEN

	**MIS**	**OPEN**	***p*-value**
	***N* = 3,601**	***N* = 5,863**	
Age in year, median (IQR)	70 (63.0, 77.0)	69 (61.5, 76.5)	0.316
missing	0 (0)	2 (0.03)	
Male sex	2,081 (57.8)	3,690 (62.9)	< 0.001
ASA^a^ I-II/ASA^a^ III-V,	2,763 (76.7)/782 (21.7)	4,180 (71.3)/1607 (27.4)	< 0.001
missing	56 (1.6)	76 (1.3)	
BMI^b^, median (IQR)	25.5 (22.9, 28.1)	25.6 (28.3, 33.7)	0.512
missing	51 (0.1)	117 (1.9)	
Tumour height^c^			0.247
Low, < 5 cm	744 (20.7)	1,218 (20.8)	
Middle, 5–10 cm	1,295 (36.0)	2,176 (37.1)	
High, 10-15 cm	1,539 (42.7)	2,390 (40.8)	
missing	23 (0.6)	79 (1.3)	
cT stage^d^			< 0.001
T1-T2	1,152 (32.0)	1,343 (22.9)	
T3	1,975 (54.8)	3,037 (51.8)	
T4	399 (11.1)	1,294 (22.1)	
missing	75 (2.1)	189 (3.2)	
cN stage^e^			< 0.001
N0	1,573 (43.7)	2,130 (36.3)	
N1-N2	1,971 (54.7)	3,492 (59.6)	
missing	57 (1.6)	241 (4.1)	
cTNM^f^			< 0.001
I	809 (22.5)	886 (15.1)	
II	675 (18.7)	1,073 (18.3)	
III	1,817 (50.6)	3,007 (51.3)	
IV	199 (5.5)	599 (10.2)	
missing	101 (2.8)	298 (5.1)	
Pre-operative treatment			< 0.001
No treatment	1,327 (36.8)	1,754 (29.9)	
Radiation only	1,682 (46.7)	2,406 (41.0)	
Radiochemotherapy	571 (15.9)	1,638 (27.9)	
Chemotherapy only	16 (0.4)	55 (0.9)	
missing	5 (0.1)	10 (0.2)	

Data expressed as No. with percentage given as percentage of column displayed within brackets unless otherwise indicated

^a^American Society of Anesthesiologists classification of physical health of the surgical patient

^b^BMI is calculated as weight in kilograms divided by height in meters square

^c^Tumour height defined in cm from the anal verge measured with rigid rectoscope

^d^Stage describing the depth of tumour invasion

^e^Stage describing nodal involvement

^f^Clinical classification of malignant tumors

**Table 2:** Intraoperative and pathological characteristics of patients who were diagnosed with rectal cancer in Sweden 2012–2018 and underwent surgical resection MIS vs OPEN

	**MIS**	**OPEN**	***p*-value**
	***N* = 3,601**	***N* = 5,863**	
Surgical resection			< 0.001
Anterior resection	1,928 (53.5)	2,791 (47.6)	
Abdominoperineal resection	1,388 (38.5)	2,260 (38.5)	
Hartmann's procedure	284 (7.9)	812 (13.8)	
Diverting ileostomy^a^	1,420 (73.7)	2,229 (80.0)	< 0.001
MIS converted to open	523 (14.5)	n/a	
missing	6 (0.2)	n/a	
Operating time in minutes, median (IQR)	333.5 (252.5, 414.5)	277.0 (190.5, 363.5)	< 0.001
missing	22 (0.6)	61 (1.0)	
Intraoperative bleeding in ml, median (IQR)	100 (25.0, 175.0)	350(300.0, 400.0)	< 0.001
missing	104 (2.9)	113 (1.9)	
Perforation	132 (3.7)	301 (5.1)	0.001
perforations close to tumour	61 (1.7)	158 (2.7)	0.134
T-stage (yp/pT^b^)			< 0.001
T0	124 (3.5)	224 (3.9)	
T1	363 (10.2)	435 (7.5)	
T2	1,157 (32.6)	1,560 (26.9)	
T3	1,765 (49.7)	3,011 (52.0)	
T4	140 (3.9)	562 (9.7)	
missing	52 (1.4)	71 (1.2)	
Nodal status (yp/pN^c^)			0.001
N0	2,237 (62.1)	3,459 (59.0)	
N1	948 (26.3)	1,601 (27.3)	
N2	360 (10.0)	724 (12.3)	
missing	56 (1.5)	79 (1.3)	
yp/pTNM^d^			< 0.001
I	1,095 (30.4)	1,518 (25.9)	
II	868 (24.1)	1,572 (26.8)	
III	1,144 (31.7)	1,934 (33.0)	
IV	139 (3.9)	506 (8.6)	
missing	355 (9.8)	333 (5.7)	
Tumour deposit	475 (13.2)	921 (15.7)	< 0.001
missing	148 (4.1)	470 (8.0)	
Vascular invasion – yes	920 (25.5)	1,514 (25.8)	0.927
missing	145 (4.0)	157 (2.7)	
Perineural invasion – yes	671 (18.6)	1,116 (19.0)	0.700
missing	146 (4.0)	213 (3.6)	
Cancer differentiation – high grade	440 (12.2)	813 (13.9)	0.001
missing	287 (8.0)	754 (12.9)	

Data expressed as No. with percentage given as percentage of column displayed within brackets unless otherwise indicated

^a^Percentage of anterior resection with diverting ileostomy

^b^Stage describing the size of the tumour

^c^Stage describing nodal involvement

^d^Pathological classification of malignant tumors

### Pathology outcomes

A positive CRM, i.e., less than 1 mm was reported in 135 patients (3.8%) of patients who had undergone MIS compared to 315 patients (5.4%) of patients who had undergone OPEN with a risk difference of -1.6% (95% CI -1.623, -1.622, *P* < 0.001) (Table 3). The upper limit of the confidence interval was below the pre-determined non-inferiority margin of 2.4%. A positive margin (R1) was reported in 2.8% of patients in the MIS group compared to 4.4% in the OPEN group with a risk difference of -1.6% (95% CI -1.649, -1.633, *P* < 0.001) (Table 3). This confidence interval was below the non-inferiority margin of 4%. Neither of the confidence intervals included 0 and both *P*-values were < 0.001 indicating superiority for MIS compared to OPEN. However, in both the unweighted and weighted adjusted regression analyses there was no statistically significant difference in positive CRM (*P* = 0.101 and *P* = 0.095) (Table 4). Neither was there a statistically significant difference in R1 resections in the adjusted unweighted and weighted analyses (*P* = 0.083 and *P* = 0.061) when comparing the two groups (Table 4). No significant between-group differences in rates of positive DRM or in rate of specimens containing less than 12 lymph nodes was seen in the adjusted analyses (Table 4).

**Table 3:** Primary and secondary outcomes for patients who were diagnosed with rectal cancer in Sweden 2012–2018 and underwent surgical resection MIS vs OPEN. Risk difference (MIS-OPEN) analyses and non-inferiority analyses

	**MIS**	**OPEN**		
	***N* = 3,601**	***N* = 5,863**	**Risk Difference** **(95% CI)**	***p*-value**
CRM < 1mm^a^, % (95% CI)	3.75 (3.41, 4.76)	5.37 (5.28, 6.54)	-1.623 (-1.623, -1.622)	< 0.001
missing, N (%)	294 (8.2)	532 (9.1)		
R1^b^, % (95%CI)	2.78 (2.35, 3.47)	4.42(4.04, 5.14)	-1.641 (-1.649, -1.633)	< 0.001
missing, N (%)	163 (4.5)	220 (3.7)		

^a^Circumferential resection margin

^b^Classification on resection margin: R0 no malignant cells at resection margin and R1 microscopic residual tumor

**Table 4:** Primary and secondary outcomes for patients who were diagnosed with rectal cancer in Sweden 2012–2018 and underwent surgical resection MIS vs OPEN. Unadjusted and adjusted results including unweighted and weighted analysis

	**MIS**	**OPEN**	**Unadjusted**		**Unweighted Regression**		**Weighted Regression**	
	***N* = 3,601**	***N* = 5,863**	**OR (95% CI)**	***p*-value**	**OR (95% CI)**	***p*-value**	**OR (95% CI)**	***p*-value**
CRM^a^ < 1 mm	135 (3.8)	315 (5.4)	0.678 (0.551, 0.833)	< 0.001	0.817 (0.641, 1.041)	0.101	0.805 (0.624, 1.038)	0.095
missing	294 (8.2)	532 (9.1)						
R1^b^	100 (2.8)	259 (4.4)	1.606 (1.270, 2.031)	< 0.001	1.268 (0.969, 1.660))	0.083	1.318 (0.987, 1.761)	0.061
missing	163 (4.5)	220 (3.7)						
DRM^c^ < 1 mm	9 (0.2)	12 (0.2)	1.236 (0.520, 2.937)	0.631	1.185 (0.420, 3.342)	0.748	1.108 (0.405, 3.027)	0.842
missing	279 (7.7)	391 (6.7)						
30-day mortality	19 (0.5)	70 (1.2)	0.439 (0.264, 0.730)	0.002	0.490 (0.276, 0.868)	0.014	0.513 (0.287, 0.920)	0.025
missing	0 (0)	0 (0)						
90-day mortality	37 (1.0)	120 (2.0)	0.497 (0.343, 0.720)	< 0.001	0.488 (0.320, 0.744)	0.001	0.487 (0.318, 0.744)	0.001
missing	0 (0)	0 (0)						
Anastomotic leak	166 (4.6)	231 (3.9)	0.115 (0.961, 1.445)	0.115	1.095 (0.862, 1.393)	0.456	0.968 (0.771, 1.214)	0.776
Re-operation 30-days	327 (9.08)	532 (9.07)	0.990 (0.866, 1.157)	0.990	1.054 (0.894, 1.244)	0.530	0.940 (0.794, 1.113)	0.473
missing	23 (0.6)	37 (0.6)						
Re-admission 30-days	807 (22.4)	1,545 (26.3)	0.807 (0.732, 0.890)	< 0.001	0.770 (0.690, 0.860)	< 0.001	0.855 (0.762, 0.959)	0.008
missing	0 (0)	0 (0)						
Re-admission 90-days	1,235 (34.3)	2,332 (39.8)	0.790 (0.725, 0.862)	< 0.001	0.800 (0.725, 0.883)	< 0.001	0.853 (0.771, 0.944)	0.002
missing	0 (0)	0 (0)						
< 12 lymph nodes in specimen	423 (11.7)	791 (13.5)	1.172 (1.033, 1.330)	0.014	1.047 (0.903, 1.213)	0.543	1.092 (0.937, 1.273)	0.257
missing	66 (1.8)	108 (1.8)						

Data expressed as No. with percentage given as percentage of column displayed within brackets unless otherwise indicated

^a^Circumferential resection margin

^b^R1 is defined as tumour cells present at resection margin in the SCRCR

^c^Distal resection margin

### Clinical outcomes

The 30- and 90-day mortality were statistically significantly lower in the MIS group (30-day; 0.5% and 90-day; 1.0%) compared to the OPEN group (30-day; 1.2% and 90-day; 2.0%) in the non-adjusted (30-day; *P* = 0.002 and 90-day; *P* < 0.001), adjusted unweighted (30-day; *P* = 0.014 and 90-day; *P* = 0.001) and weighted analyses (30-day; *P* = 0.025 and 90-day; *P* = 0.001) (Table 4). Similarly, 30- and 90-day readmission were less common in the MIS group compared to the OPEN group (Table 4). There was no significant difference in anastomotic leak, nor reoperation within 30-days comparing MIS and OPEN in the adjusted unweighted and weighted analyses (Table 4). Length of hospital stay was shorter in the non-adjusted and adjusted unweighted and weighted analyses (*P* < 0.001) following MIS (median 7 days vs 9 days) (Table 5).

**Table 5:** Secondary outcome for patients who were diagnosed with rectal cancer in Sweden 2012–2018 and underwent surgical resection MIS vs OPEN. Unadjusted and adjusted results including unweighted and weighted linear regression

	**MIS**	**OPEN**	**Unadjusted**		**Unweighted Regression**		**Weighted Regression**	
	***N* = 3,601**	***N* = 5,863**	**OR (95% CI)**	***p*-value**	**OR (95% CI)**	***p*-value**	**OR (95% CI)**	***p*-value**
Length of hospital stay in days, median (IQR)	7 (4.0, 10.0)	9 (5.0, 13.0)	-0.132 (-0.144, -0.121)	< 0.001	-0.103 (-0.116, -0.090)	< 0.001	-0.103 (-0.114, -0.092)	< 0.001
missing	29 (0.8)	34 (0.6)						

Data expressed as median and interquartile range (IQR) displayed within brackets

### Subgroup analyses – ROBOT vs OPEN

In the subgroup analyses 2014 to 2018 we found that 19.6% of patients underwent LAP, 26.5% ROBOT and 53.9% OPEN. The baseline characteristics comparing MIS vs OPEN revealed a higher proportion of male patients, higher ASA and lower tumours, similar selection have been previously reported (Table 6) [24, 25]. Patients in the OPEN group again displayed more advanced cancer stages along with other features suggestive of more aggressive tumour biology (Tables 6 and 7). ROBOT and LAP cases had less bleeding but longer operating times, the longest median operating time was in the ROBOT group (Table 7). The conversion rate was significantly higher in LAP compared to ROBOT (18.5% vs 10.7%, *P* =  < 0.001) (Table 7).

**Table 6:** Subgroup analyses. Baseline characteristics of patients who were diagnosed with rectal cancer in Sweden 2014–2018 and underwent surgical resection 2014–2018 LAP, ROBOT and OPEN

	**LAP**	**ROBOT**	**OPEN**	***p*-value**
	***N* = 1,383**	***N* = 1,867**	***N* = 3,797**	
Age in year, median (IQR)	70.0 (63.3, 76.5)	69 (62.5, 75.5))	69(62.0, 76.0)	0.316
missing	0	0	2	
Male sex	789 (57.0)	1,107 (59.2)	2,425 (63.9)	< 0.001
ASA^a^ I-II/ASA^a^ III-V,	1,075 (77.7)/289 (20.9)	1,406 (75.3)/427 (22.9)	2,594 (68.3)/1,135 (30.0)	< 0.001
missing	20 (1.4)	34 (1.8)	68 (1.8)	
BMI^b^, median (IQR)	25.5 (22.9, 28.1)	25.5 (23.0, 28.1)	25.7 (23.0, 28.4)	0.512
missing	14 (1.0)	23 (1.2)	56 (1.5)	
Tumour height^c^				0.247
Low, < 5 cm	267 (19.3)	386 (20.7)	800 (21.1)	
Middle, 5–10 cm	486 (35.1)	703 (37.7)	1,447 (38.1)	
High, 10-15 cm	619 (44.8)	769 (41.1)	1,503 (39.6)	
missing	12 (0.9)	9 (0.5)	47 (1.2)	
cT stage^d^				< 0.001
T1-T2	459 (33.2)	575 (30.8)	830 (21.9)	
T3	760 (54.9)	1,025 (54.9)	1,919 (50.5)	
T4	135 (9.8)	232 (12.4)	958 (25.2)	
missing	30 (2.2)	35 (1.9)	90 (2.4)	
cN stage^e^				< 0.001
N0	606 (43.8)	797(42.7)	1,297 (34.1)	
N1-N2	764 (55.2)	1,040 (55.7)	2,409 (63.4)	
missing	14 (1.0)	30 (1.6)	91 (2.4)	
cTNM^f^				< 0.001
I	315 (22.7)	404 (21.6)	553 (14.6)	
II	253 (18.3)	352 (18.8)	643 (16.9)	
III	699 (50.5)	961 (51.5)	2,051 (54.0)	
IV	82 (5.9)	101 (5.4)	432 (11.4)	
missing	35 (2.5)	49 (2.6)	118 (3.1)	
Pre-operative treatment				< 0.001
No treatment	512 (37.0)	673 (35.9)	1,090 (28.7)	
Radiation only	711 (51.4)	799 (42.8)	1,436 (37.8)	
Radiochemotherapy	154 (11.1)	382 (20.4)	1,216 (32.0	
Chemotherapy only	5 (0.4)	10 (0.5)	45 (1.2)	
missing	2 (0.1)	3 (0.2)	10 (0.3)	

Data expressed as No. with percentage given as percentage of column displayed within brackets unless otherwise indicated

^a^American Society of Anesthesiologists classification of physical health of the surgical patient

^b^BMI is calculated as weight in kilograms divided by height in meters square

^c^Tumour height defined in cm from the anal verge measured with rigid rectoscope

^d^Stage describing the depth of tumour invasion

^e^Stage describing nodal involvement

^f^Clinical classification of malignant tumors

**Table 7:** Intraoperative and pathological characteristics of patients who were diagnosed with rectal cancer in Sweden 2012–2018 and underwent surgical resection MIS vs OPEN

	**LAP**	**ROBOT**	**OPEN**	***p*-value**
	***N* = 1,383**	***N* = 1,867**	***N* = 3,797**	
Surgical resection				< 0.001
Anterior resection	730 (52.8)	1,018 (54.5)	1,704 (44.9)	
Abdominoperineal resection	536 (38.8)	696 (37.3)	1,529 (40.3)	
Hartmann's procedure	117 (8.4)	153 (8.2)	564 (14.9)	
Diverting ileostomy^a^	526 (72.0)	781 (76.7)	1,346 (79.0)	< 0.001
MIS converted to open	256 (18.5)	201 (10.7)	n/a	< 0.001
Missing	4 (0.3)	2 (0.1)	n/a	
Operating time in minutes, median (IQR)	309.0 (229.0, 389.0)	354.0 (275.5, 432.5)	288.0 (197.0, 379.0)	< 0.001
Missing	13 (0.9)	7 (0.4)	18 (0.5)	
Intraoperative bleeding in ml, median (IQR)	100.0 (15.0, 185.0)	100.0 (25.0, 175.0)	350.0 (125.0, 575.0)	< 0.001
Missing	35 (2.5)	37 (2.0)	72 (1.9)	
Perforation	42 (3.0	76 (4.1)	200 (5.3)	0.001
Perforations close to tumour	14 (1.0)	39 (2.1)	110 (2.9)	0.023
T-stage (yp/pT^b^)				< 0.001
T0	37 (2.7)	75 (4.0)	154 (4.0)	
T1	140 (10.1)	188 (10.0)	271 (7.1)	
T2	426 (30.8)	605 (32.4)	981 (25.8)	
T3	713 (51.5)	885 (47.4)	1,910 (50.3)	
T4	49 (3.5)	84 (4.5)	418 (11.0)	
missing	19 (1.4)	30 (1.6)	63 (1.6)	
Nodal status (yp/pN^c^)				0.022
N0	864 (62.4)	1,138 (60.9)	2,222 (58.5)	
N1	350 (25.3)	519 (27.8)	1,066 (28.0)	
N2	151 (10.9)	177 (9.4)	443 11.6)	
missing	19 (1.4)	33 (1.8)	66 (1.7)	
yp/pTNM^d^				< 0.001
I	402 (29.1)	565 (30.3)	946 (24.9)	
II	240 (17.3)	436 (23.3)	1,015 (26.7)	
III	417 (30.1)	625 (33.5)	1,233 (32.5)	
IV	55 (4.0)	71 (3.8)	336 (8.8)	
Missing	170 (12.3)	170 (9.1)	267 (7.0)	
Tumour deposit	165 (11.9)	276 (14.3)	652 (17.1)	< 0.001
Missing	45 (3.2)	59 (3.1)	179 (4.7)	
Vascular invasion—yes	303 (21.9)	550 (29.4)	1,029 (27.1)	< 0.001
Missing	53 (	81 (4.3)	129 (3.4)	
Perineural invasion—yes	263 (19.0)	353 (18.9)	800 (21.1)	0.110
Missing	51 (3.7)	78 (4.2)	129 (3.4)	
Cancer differentiation—high grade	159 (11.5)	258 (13.8)	526 (13.8)	0.017
Missing	81 (5.9)	156 (8.3)	389 (10.2)	

Data expressed as No. with percentage given as percentage of column displayed within brackets unless otherwise indicated

^a^Percentage of low anterior resection with diverting ileostomy

^b^Stage describing the depth of tumour invasion

^c^Stage describing nodal involvement

^d^Pathological classification of malignant tumors

Comparison of ROBOT to OPEN revealed no statistically significant difference in CRM < 1 mm (*P* = 0.840) or in R1 resections (*P* = 0.738) in the adjusted analyses. Neither was there a difference in the pathology assessment with regard to DRM < 1 mm or a specimen including < 12 resected lymph nodes (Table 8). However, 90-day mortality and 30 and 90-day re-admissions were significantly higher in the unadjusted and adjusted analyses following OPEN compared to ROBOT (Table 8). No difference was noted in the adjusted analyses for 30-day mortality, anastomotic leak, reoperation within 30 days between the two groups but LOS was shorter following ROBOT versus OPEN (7 vs 9 days, *p* < 0.001) (Table 9).

**Table 8:** Subgroup analyses. Primary and secondary outcomes for patients who were diagnosed with rectal cancer in Sweden 2014–2018 and underwent surgical resection 2014–2018 LAP, ROBOT and OPEN. Unadjusted and adjusted results comparing ROBOT with OPEN

	**LAP**	**ROBOT**	**OPEN**	**Unadjusted**		**Adjusted**	
	***N* = 1,383**	***N* = 1,867**	***N* = 3,797**	**OR (95% CI)**	***P*-value**	**OR (95% CI)**	***p*-value**
CRM < 1mm^a^	41 (3.0)	81 (4.3)	220 (5.8)	1.377 (1.060, 1.789)	< 0.016	1.032 (0.761, 1.398)	0.840
missing	105 (7.6)	152 (8.1)	355 (9.3)				
R1, n**	33 (2.4)	58 (3.1)	168 (4.4)	0.693 (0.512, 0.940)	0.018	0.944 (0.673, 1.324)	0.738
missing	75 (5.4)	83 (4.4)	163 (4.2)				
DRM < 1mm^a^	2 (0.1)	6 (0.3)	7 (0.2)	0.572 (0.192, 1.704)	0.316	0.627 (0.1784, 2.259)	0.475
missing	107 (7.7)	139 (7.4)	277 (7.2)				
30-day mortality	9 (0.7)	8 (0.4)	39 (1.0)	2.412 (1.125, 5.171)	0.024	2.094 (0.896, 4.896)	0.088
missing	0 (0)	0 (0)	0 (0)				
90-day mortality	17 (1.2)	17 (0.9)	79 (2.0)	2.312 (1.365, 3.917)	0.002	2.106 (1.174, 3.778)	0.013
missing	0 (0)	0 (0)	0 (0)				
Anastomotic leak	51 (3.7)	104 (5.6)	152 (4.0)	0.707 (0.547, 0.913)	0.008	0.772 (0.579, 1.030)	0.079
Re-operation 30-days	123 (8.9)	169 (9.0)	331 (8.7)	0.960 (0.791, 1.166)	0.682	0.914 (0.739, 1.130)	0.404
missing	4 (0.2)	17 (0.9)	37 (1.0)				
Re-admission 30-days	256 (18.5)	449 (24.0)	1,086 (28.6)	1.265 (1.114, 1.437)	< 0.001	1.240 (1.080, 1.423)	0.002
missing	0 (0)	0 (0)	0 (0)				
Re-admission 90-days	421 (30.4)	668 (35.8)	1,587 (41.7)	1.289 (1.149, 1.1445)	< 0.001	1.213 (1.071, 1.373)	0.002
missing	0 (0)	0 (0)	0 (0)				
< 12 lymph nodes in specimen	166 (12.0)	203 (10.9)	473 (12.5)	0.853 (0.716, 1.016)	0.074	0.947 (0.774, 1,158)	0.595
missing	24 (18.5)	34 (1.8)	85 (2.3)				

Data expressed as No. with percentage given as percentage of column displayed within brackets

^a^Circumferential resection margin

^**^Classification on resection margin: R0 no malignant cells at resection margin and R1 microscopic residual tumor

**Table 9:** Subgroup analyses. Secondary outcome for patients who were diagnosed with rectal cancer in Sweden 2014–2018 and underwent surgical resection LAP, ROBOT and OPEN. Unadjusted and adjusted comparing ROBOT with OPEN

	**LAP**	**ROBOT**	**OPEN**	**Unadjusted**		**Adjusted**	
	***N* = 1,383**	***N* = 1,867**	***N* = 3,797**	**OR (95% CI)**	***P*-value**	**OR (95% CI)**	***P*-value**
Length of hospital stay in days, median (IQR)	7.0 (4.0, 10.0)	7.0 (4.0. 10.0)	9.0 (5.0, 13.0)	-0.147 (-0.162, -0.131)	< 0.001	-0.127 (-0.143, -0.111)	< 0.001
missing	5 (0.4)	19 (1.0)	32 (0.8)				

Data expressed as median and interquartile range (IQR) displayed within brackets

## Discussion

This population based study indicated that MIS is non-inferior to OPEN for adequacy of cancer resection in routine healthcare. This is found partly through no difference in positive CRM and R1 in the adjusted unweighted and weighted analyses. MIS also demonstrated a significantly lower 30- and 90-day mortality and re-admission rates compared to OPEN, both in the adjusted unweighted and weighted analyses. Previously well recognized advantages of MIS over OPEN were confirmed including less bleeding and shorter hospital stay.

Positive CRM and positive DRM are both recognized as important prognosticators for local recurrence following rectal cancer resection [26–28]. The rates of positive CRM and positive DRM in this study are similar to rates reported by the most recent randomised controlled trials and lower compared to rates previously reported in large cohort and population based studies [14, 28, 29]. In comparison to the randomised trials, this population based study included a smaller proportion of low tumours (20.7% MIS and 20.8% OPEN) which facilitates the possibility of achieving an adequate cancer resection. On the other hand, and in contrast to randomised trials, this study also included cT4 tumours (11.1% MIS and 22.1% OPEN) which is considered a risk factor for positive resection margins. A population based study from the Netherlands reported positive CRM in as many as 13.9% of TME resections for cT4 rectal cancers [30]. Other known risk factors for a positive margin include previous radiochemotherapy, T3 tumours, N stage 1–2, APR and high BMI [31, 32]. There were similar percentages of cT3 tumours and cN stage 1–2 in our study, but a higher proportion of preoperative radiation or radiochemotherapy in all groups when comparing with the randomised trials. A higher proportion in our study have undergone APR, though this study included by comparison a smaller proportion of low tumours. This is in keeping with previously reported higher rates of APR in Sweden compared to the Netherlands [33]. Furthermore, reflective of the Swedish population, this study reports a relatively low median BMI when compared to many other western populations. This population based study mirrors the real world and the distribution of disease stage, tumour height and the use of preoperative radiation and chemoradiation reflects this. However, the distribution of these important characteristics differ from those seen in non-population based studies including randomised controlled trials which makes direct comparison of outcomes difficult.

The 30- and 90-day mortality is lower than previously described in cohort studies and population based studies and similar to rates reported in randomised controlled trials [1, 4, 9, 29, 34, 35]. The explanation for this is likely multifactorial, factors that might play a role are: the presence of a gradual centralisation process, appropriate patient selection for surgery and the proportion of patients undergoing resection [36–38]. Furthermore, patients’ comorbidities as well as their age have also been shown influence short term mortality. Sweden has previously been demonstrated to perform a higher proportion of rectal cancer resections when compared to England, Denmark and Norway, especially in the older population [39]. It is also worth mentioning that most elective rectal cancer resections in Sweden are performed by specialized colorectal surgeons. The favourable morbidity and mortality rates following MIS compared to OPEN are in accordance with those previously reported in large cohort and population based studies [15, 34, 40, 41]. Our subgroup analyses comparing ROBOT and OPEN found similar outcomes for ROBOT as for MIS including decreased 90-day mortality and decreased 30- and 90-day readmission. A decreased 30- and 90-day readmission in the MIS group has been suggested by previous population based studies [40, 41].

The rate of diverting stomas are notably higher than those reported in other studies, which is in keeping with results previously reported comparing Swedish and Dutch data.

This could in part be explained by the frequent use of radiation in accordance with the Swedish national treatment guidelines for rectal cancer. Also, many centers in Sweden have accepted the results from the RECTODES trial that diverting loop stomas are reported to decrease the rate of symptomatic anastomotic leakage [42, 43]. A higher rate of conversion in the MIS group when compared to the randomized controlled trials was also found, with lower figures noted in the ROBOT group. The rate of conversion rate is used to assess the MIS learning curve, and ROBOT is considered to have a shorter learning curve compared to LAP. This could in part explain the differences between the two groups, despite ROBOT being introduced at a later stage. The centralisation for surgical treatment of rectal cancer in Sweden started in the early 2000s, though, only half of all rectal resections are performed in high volume hospitals with a yearly volume of around 50 cases, which could in part explain the higher conversion rate [33, 37]. The number of resections per surgeon is also known to influence conversion rate, however, this information is not readily available in the SCRCR. Nevertheless, this study exhibits a high proportion of adequate cancer resection and favourable short-term outcomes, indicating a safe implementation of MIS in Sweden.

Recognised drawbacks with MIS include longer operating time and higher costs for the health care sector although without a cost-difference in long- term societal perspective [44]. Studies have however invariably reported higher hospital costs related to robotic assisted laparoscopic surgery when compared to conventional laparoscopic surgery [45, 46]. We have not evaluated the health economic aspects in this study but can confirm longer operating times.

Strengths of this study include the population based setting and the combination of two high quality registers including close to all patients who have undergone curative surgery for rectal cancer in Sweden 2012–2018 with a 90 day follow up. Another strength is the consistency of the results following two different statistical methods indicating the robustness of the findings.

Limitations of this study are similar to those of other population-based register studies including the potential for selection bias and residual confounding. The indications for the choice of MIS or OPEN surgery were not available. However, the use of different statistical models demonstrating comparable results may have reduced the potential problem of residual confounding.

## Conclusions

In conclusion this population based study indicated that MIS is non-inferior to OPEN for rectal cancer with regard to adequacy of cancer resection in routine healthcare. It also demonstrated favourable short-term outcomes including significantly less bleeding, shorter hospital stay lower and lower 30- and 90-day mortality and re-admission rate. This study support that MIS for rectal cancer is a safe oncologic procedure when performed by experienced surgeons in routine health care.

## Data Availability

The datasets generated and analysed during the current study are from the National Patient Registry and the Swedish Colorectal Cancer Registry under license for the current study. Data are available from the corresponding author with the permission of National Patient Registry (https://www.socialstyrelsen.se/en/) and the Swedish Colorectal Cancer Registry (https://scrcr.se).

## Abbreviations

CRM: Circumferential resection margin
R1: Positive resection margin
LOS: Length of stay
MIS: Minimally invasive surgery
OPEN: Open surgery
ROBOT: Robotic assisted laparoscopic surgery
SCRCR: Swedish Colorectal Cancer Registry
NPR: National Patient Register
DRM: Distal resection margin
ASA: American Society of Anesthesiologists classification
BMI: Body mass index
cTNM: Clinical tumour stage
AR: Anterior resection
APR: Abdominoperineal resection
LAP: Laparoscopic surgery
